# Evaluation of Factors Impacting the Efficacy of Single or Combination Therapies of Valproic Acid, Carbamazepine, and Oxcarbazepine: A Longitudinal Observation Study

**DOI:** 10.3389/fphar.2021.641512

**Published:** 2021-05-04

**Authors:** Qilin Peng, Mubai Ma, Xurui Gu, Yani Hu, Boting Zhou

**Affiliations:** ^1^ Department of Pharmacy, Xiangya Hospital, Central South University, Changsha, China; ^2^ The Hunan Institute of Pharmacy Practice and Clinical Research, Changsha, China

**Keywords:** anti-epileptic drugs, valproic acid, carbamazepine, oxcarbazepine, serum concentrations, AED efficacy

## Abstract

**Objective:** This study aimed to determine the efficacy and clinical factors related to the pharmacodynamics of single or combination therapies of valproic acid (VPA), carbamazepine (CBZ), and oxcarbazepine (OXC), three commonly used anti-epileptic drugs (AEDs) in China.

**Methods:** The study evaluated the records of 2027 outpatients in a Changsha hospital, located in China, from December 23, 2015 to October 28, 2019. The baseline seizure frequency was assessed during the first visit. AED efficacy was determined based on the reduction in seizures from baseline at the subsequent visits. Multivariable ordinal regression analysis was used to determine the association between the clinical factors (demographic characteristics, clinical features, and medication situation) and AED efficacy. For validation, the clinical efficacies of AEDs were compared as both single agents and in combinations. Differences in adverse effect (AEs) categories were analyzed by Chi-square between AED groups.

**Results:** Records of patients receiving VPA, CBZ, and OXC were evaluated. Serum concentrations of VPA and CBZ is significantly correlated with efficacy (OR 1.030 [1.024–1.037], *p* < 0 0.0001; OR 1.250 [1.146–1.63], *p* < 0.0001, respectively) and OXC efficacy correlated to the serum concentration of the metabolite 10,11-dihydro-10-hydroxy-carbazepine (monohydroxy derivative, MHD) serum concentrations (OR 1.060 [1.031–1.089], *p* < 0.0001). Significant differences existed between females and males in VPA efficacy (OR 1.318 [1.033–1.682], *p* = 0.027). After validation, VPA, in combination with OXC (OR 1.93 [1.38–2.70], *p*<0.001), or with VGB (Vigabatrin) (OR 2.36 [1.38–2.70], *p* = 0.002), showed significantly better efficacy than as a single agent. OXC efficacy was also affected by the duration of epilepsy (OR 0.965 [0.946–0.984], *p* < 0.001). Additionally, the efficacies of OXC and VPA were also affected by the seizure type. Seizure reduction improved significantly with an increasing number of pharmacists’ educations in the first three visits period. There were no differences in AEs incidence among these 3 AEDs except for Psychiatric (0.02) and nervous system disorders (0.0001).

**Conclusion:** Serum concentrations of VPA and CBZ may positively affect their efficacies, while OXC efficacies are correlated to MHD serum concentrations. The efficacy of VPA was higher in females compared to males. VPA-OXC and VPA-VGB combinations had higher efficacies compared to monotherapy. Besides, OXC efficacy is probably reducing by the duration of epilepsy. Additionally, VPA efficacy for focal or generalized seizures is superior to mixed-type seizures. OXC was more effective for focal seizures compared to mixed-type ones. Education provided by pharmacists improved the seizures to some extent, and there were no significant differences between most categories of adverse effects for the investigated AEDs.

## Introduction

Epilepsy is one of the most common chronic neurological disorders, requiring continuous attention. Accounting for 1% of the world disease burden (Kwon et al., 2011), it has been ranked as the second most burdensome neurological disorder worldwide in terms of disability-adjusted life years (Murray et al., 2012). There are 45.9 million all-active patients with epilepsy (PWE) worldwide (Collaborators, 2019). The high prevalence and incidence of epilepsy cause tremendous economic loss and societal burden. To date, anti-epileptic drugs (AEDs) are the primary treatment, and they control seizures without normalizing the potential neuropathological process. As a result, PWE often requires life-long AED treatment (Chen et al., 2018). However, unpredicted seizures, indeterminable efficacy of AEDs, and their unexpected adverse effects (AEs) can markedly affect the patient’s quality of life. Although the efficacy of AEDs is widely acknowledged to correlate with their serum concentrations, there are more potential confounding factors such as associated diseases and other co-medications (Zaccara and Perucca, 2014). The factors that influence the efficacy of AEDs and their related AEs have not been fully understood. While the pharmacokinetics of AEDs is well understood, further studies are needed to determine the factors related to their efficacy. According to a recent meta-analysis there are limited studies that characterize the relationship between the levels of AEDs and their therapeutic/toxic effects (Methaneethorn, 2018). Investigating the parameters that influence the therapeutic effects and AEs of AEDs may be beneficial in improving the clinical management of epilepsy, including drug selection and replacement.

There is no consensus whether AEDs polytherapy improve the efficacy compared to monotherapy since 1981 (Reynolds et al., 1981). Growing evidence has shown that unrestrained polytherapy could even exacerbate epilepsy in some patients (Reynolds et al., 2012). Since certain combinations have been proved to be more effective than others, generalizations about the poor effectiveness of polytherapy cannot be made (Deckers, 2002). However, one study demonstrated no efficacy differences between efficacy of CBZ-monotherapy and its combination with VPA (Deckers et al., 2001), Another study, which involved 148 patients, also found that there is no significant improvement of the comparative efficacy of individual AED with other regimens except for LTG-VPA (not include OXC) (Poolos et al., 2012), Currently, there is no reliable answer as to whether PWE would benefit more from a monotherapy or an AED combination. Since the studies of synergism with a particular AED combination are limited, further large-sample studies are still needed to explore the impact of the increasing range of newer agents on clinical outcomes (Stephen and Brodie, 2012). As plentiful publications have valided the higher incidence of AEs of polytherapy than a single has, we did not focus on that. Since recent reports have claimed that the AEs of second-generation AEDs as a monotherapy or combination therapy is comparable to conventional AEDs (Kumar et al., 2020), we compared the AEs occurrences in patients with 3 AEDs monotherapy in order to verify this perspective.

Valproic acid (VPA), carbamazepine (CBZ), and oxcarbazepine (OXC) are among the most widely used AEDs (Sitges et al., 2007). VPA is the first drug of choice for patients with generalized and unclassifiable epilepsy (French, 2007), while CBZ is recommended as the first-line AED for focal seizures (Marson et al., 2007). OXC is well tolerated as a single agent by PWE with focal seizures (Herranz et al., 2004). As the clinical efficacy of these AEDs varies with each other, one of the purposes of this study was to determine the factors affecting the clinical efficacy of VPA, CBZ, and OXC in PWE. As recent reports have claimed that several outcomes are possible of AEDs-polytherapy, the combination may be ineffective or sometimes detrimental (Stephen and Brodie, 2012). Since the principle of anti-seizure medications is to decrease continuing seizures while minimizing AEs (Thijs et al., 2019), we also compared both efficacy in monotherapy in combinations and incidence of AEs associated with VPA, CBZ, and OXC in order to explore ideal AED-polytherapy. We believe our findings will help in choosing AEDs combination regimens for clinicians.

## Methods

### Patients

This prospective study was designed to collect information on outpatients who were first diagnosed with epilepsy at the Xiangya Hospital of Central South University. From December 23, 2015 to October 28, 2019. We enrolled 5,404 consecutive records from PWE, including seizure frequency and other clinical information. According to the International League of Epilepsy (ILAE) definitions, epilepsy is defined as the occurrence of at least two unprovoked (or reflex) seizures > 24 h apart. All patients included in our study were diagnosed with epilepsy by clinicians and neuroscientists based on medical history and auxiliary examination. Information on baseline seizure frequency was obtained during the first visit from the patients directly or from those who witnessed the initial seizures (Kwan and Brodie, 2000), After that, the participants/guardians were asked to keep a seizure diary. During every subsequent visit, our pharmacists recorded detailed information based on the pharmacist-patient encounters. We also provided pharmacy service to the patients, details of which are described in our previous study (Ma et al., 2019). Follow-up surveys were conducted untill October 2019 using a form to record the patient-reported outcomes (PROs). The study was approved by the Medical Ethics Committee of the Xiangya Hospital, Central South University (approval number 2019020078). Informed consent to participate in the study was obtained from all patients/guardians.

Since the study aimed to analyze factors affecting the efficacy of AEDs, we excluded the records of patients who 1) exhibited poor medication adherence as determined by a simplified medication adherence questionnaire (SMAQ) with detailed criteria used to assess the adherence being described in our previous study (Ma et al., 2019), 2) received non-drug therapies such as surgery, electrical stimulation or ketogenic diet (Ricart, 2011), 3) were treated with a combination of AEDs other than those reported in Table 1, or other agents such as herbal medicine (Samuels et al., 2008), 4) had unevaluable information on AED efficacy, such as undetermined seizure frequency/nonepileptic seizures, or incomplete seizure diaries, 5) were in the phase of AED reduction/switching, and 6) had seizures as a result of alcohol or stimulating external factors.

**TABLE 1:** Clinical characteristics of the study participants.

	Number (range)		
Characteristics	VPA	CBZ	OXC
Records	1,049	309	669
Sex			
Male	644	200	392
Female	405	109	277
Age, yr	8.6 (0.3–82.0)	32.3 (7.7–81.8)	26.5 (0.8–22.8)
Height, cm	113.2 (50.0–183.0)	163.3 (100.0–185.0)	157.5 (75.0–183.0)
Weight, kg	25.5 (6.5–95.0)	62.4 (25.0–120.0)	57.4 (10.0–84.6)
Daily dosage, g	0.5 (0.08–4.5)	0.5 (0.02–1.8)	0.8 (0.02–3.15)
Duration of epilepsy, yr	3.6 (0.06–42.1)	13.0 (0.06–43.9)	7.0 (0.1–63.0)
Duration of this AED, yr	1.9 (0.04–28.58)	7.9 (0.03–36.0)	2.9 (0.08–38.6)
Serum concentration, mg/L	54.7 (10.9–175.0)	6.1 (1.0–17.6)	11.4 (2.5–66.5)^※^
Administration interval, h	12.9 (6.0–24.0)	10.4 (6.0–48.0)	11.7 (8.0–24.0)
Dosage form			
Oral solution	780	0	26
Sustained release tablet	182	0	0
Ordinary tablet	87	309	643
Etiology type			
Genetic	105	20	69
Structural	185	147	234
Metabolic	19	1	5
Immune	3	0	1
Infectious	65	29	51
Unknown	671	112	309
Seizure type			
Generalized	382	183	372
Focal	390	100	183
Generalized & Focal	29	21	16
Unknown	247	5	97
AED combinations			
LEV	259	31	77
LTG	86	7	23
TPM	143	8	24
VPA	-	42	102
CBZ	30	-	3
OXC	157	1	-
VGB	55	2	0
PB + PHT	9	11	14
Other combinations			
L-CAR	53	1	9
NZP	101	3	8
Hepatic function index			
ALB, g/L	43.6 (31.1–54.5)	41.8 (30.4–53.7)	42.5 (34.1–55.0)
A/G	1.9 (0.9–3.0)	1.7 (0.9–2.7)	1.9 (0.9–3.2)
TBIL, μmol/L	6.3 (1.7–21.9)	6.7 (2.2–22.4)	6.4 (1.8–19.9)
DBIL, μmol/L	2.7 (0.3–8.7)	2.9 (0.5–9.2)	2.5 (0.7–7.7)
TBA, μmol/L	3.9 (0.3–33.6)	4.2 (0.4–31.7)	3.7 (0.3–25.8)
ALT, u/l	14.9 (1.7–215.5)	16.7 (2.9–77.8)	15.1 (1.5–80.2)
AST, u/l	31.2 (3.0–101.0)	31.5 (3.0–92.9)	29.9 (4.5–95.0)

LEV: Levetiracetam; LTG: Lamotrigine; TPM: Topiramate; PHT: Phenytoin; VPA: Valproic acid; CBZ: Carbamazepine; OXC: Oxcarbazepine; VGB: Vigabatrin; PB: Phenobarbital L-CAR: Levocarnitine; NZP: Nitrazepam; ALB: Albumin; A/G: Albumin/Globulin; Tbil: Total bilirubin; DBIL: Direct bilirubin; TBA: Total bile acid;; ALT: Alanine transaminase; AST: Aspartate aminotransferase.

※: OXC is rapidly reduced to 10,11-dihydro-10-hydroxy-carbazepine (monohydroxy derivative, MHD) *in vivo*. And it is widely validated that OXC efficacy and adverse effect of oxcarbazepine appears to be related to dose and to serum concentrations of MHD (May, Korn-Merker et al., 2003). Therefore, MHD serum concentrations were measured and analyzed in this study.

### Treatment

Every patient was provided with an individualized AED treatment based mainly on the seizure type, epilepsy syndrome, co-medications, and AEs. Generally, after the clinicians prescribed the first AED, we followed up the patients’ responses by PROs and regular therapeutic drug monitoring (TDM) to determine if the dosage needed adjustment to meet the AED therapeutic window. The bioassay conditions have been described in our previous study (Ma et al., 2019). If unexpected severve AEs occurred at a low dosage or if the seizure control failed, an alternative AED was used (Perucca et al., 2011). In contrast, if the patients tolerated the first applied AED well and the seizure frequency was well-controlled the treatment was continued. Poor seizure control by the first AED treatment led to trying a combination therapy (Kwan and Brodie, 2006). PWE showing poor tolerance to two AEDs and thereby failing to achieve sustained seizure freedom with good medication adherence, was regarded as having drug-resistant epilepsy. In such cases, non-drug therapy was considered.

### Factors Evaluated for Anti-Epileptic Drug Efficacy

The following six factors were analyzed for their influence on the efficacy of AEDs: 1) demographic characteristics such as sex and age; 2) basic clinical features, such as weight and height; 3) medication history, including type, form, dosage, interval, and combinations; 4) serum biochemical index, mainly including serum concentrations of AEDs (all of them were pre-dose trough of steady-state serum concentration) and indices of blood hepatic and renal function (Alanine transaminase, aspartate aminotransferase, creatinine, bilirubin, and albumin et al.); 5) history of diseases and other medications, including duration of epilepsy, duration of AED administration; and 6) types of epilepsy and seizures. As the conditions of patients continuously changed due to aging and adjustments in the therapeutic regimen, their records were also evaluated continuously. Complications from epilepsy and AEs of the AEDs were also considered rather than quantified.

### Definitions

We classified epilepsy based on two criteria. As for its etiological classification, the history of epilepsy (injuries, anoxia, cerebral tumor, infection, and family history) was retrospectively evaluated. Additionally, the results of magnetic resonance imaging (MRI) and genetic testing were also taken into consideration. Based on etiology, epilepsy was classified as genetic, structural, metabolic, immune, infectious, or unknown. Based on seizure syndromes and electroencephalogram (EEG), seizure type was categorized as focal, generalized, generalized and focal, or unknown (Scheffer et al., 2016). AED efficacy was categorized based on the percent reduction in seizures from baseline. The efficacy was defined as 1st to 5th levels if the reduction was < 25%, 25–49%, 50–74%, 75–99%, and 100%, respectively (Nabbout et al., 2019). A 100% reduction in seizures or freedom from seizures was defined as no seizures for the previous 12 months or longer (Glauser et al., 2013).

### Statistical Methods

The Pearson χ2 test was performed to compare the categorical data on factors. For non-parametric continuous data, the homogeneity test of variance was performed. Before factors were included in the regression analysis, one-Way Analysis of Variance (ANOVA) was used to compare of homogeneity variance. The Kruskal-Wallis test was applied for comparison of heterogeneity variance as the preliminary filter for possible correlative factors. Multivariable ordinal regression was used to assess the association between filtered factors and AED efficacy. The range of odds ratio (OR) is defined by a 95% confidence interval (CI). Statistical analyses were all performed using Statistical Product and Service Solutions (SPSS) (version 25.0). Statistical significance was defined as *P* < 0.05. Mann-Whitney U test was used to analyze the differences for validation of efficacies of monotherapy of VPA, CBZ and OXC and with the combination of one specific kind of drug respectively and the polytherapy (polytherapy were regarded as a whole, which means a combination with at least one AED without separate analysis in pairwise of each AED-combinations here). Additionally, the AEs were categorized based on their mechanism of action. The Chi-square test was used to analyze the differences in the clinical efficacy between different AED groups for each of the AE categories. Statistical significance was defined as *P* < 0.05.

## Results

### Population

**FIGURE 1:**
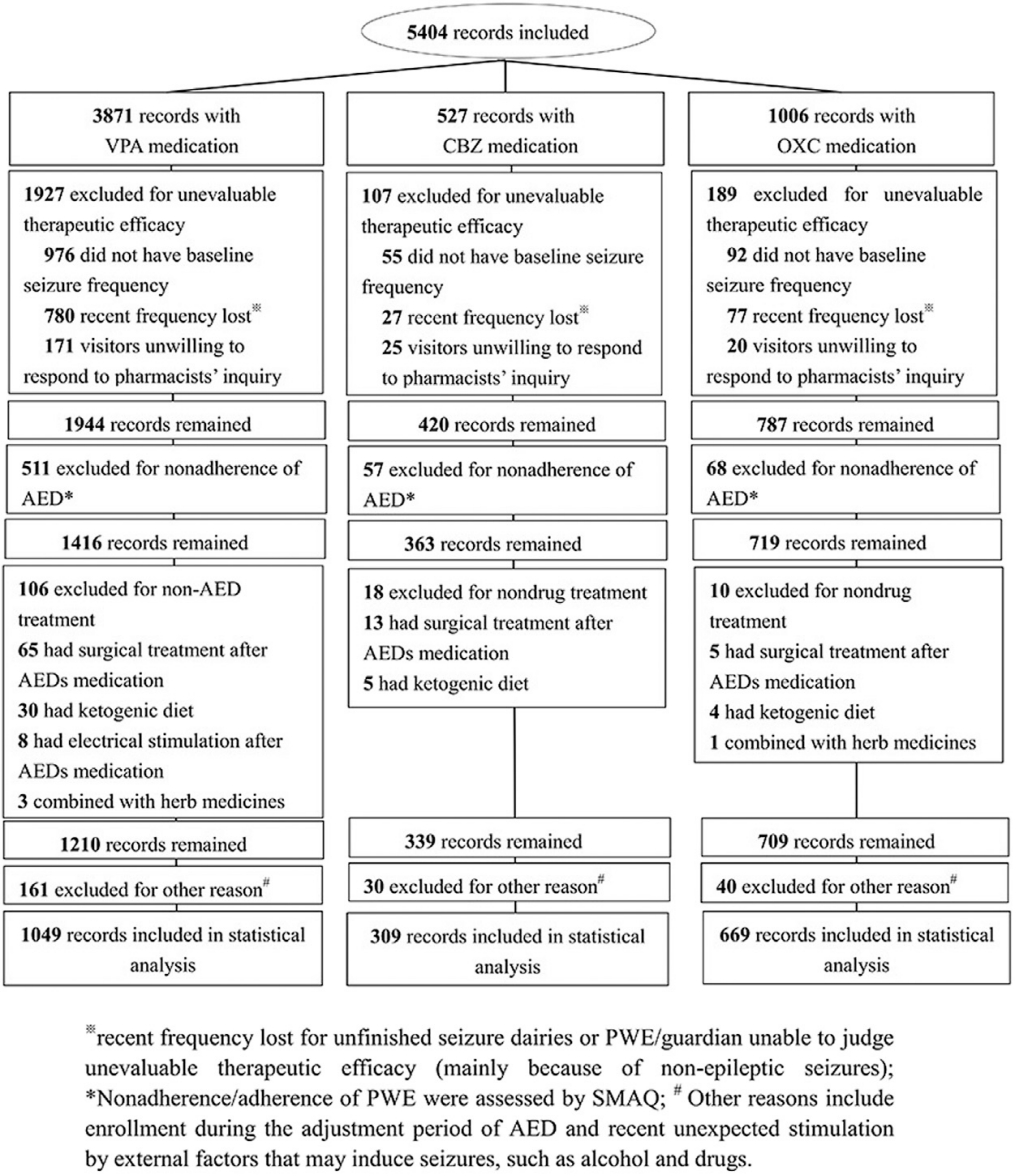
Flow diagram showing the selection of records.

A total of 2027 records of PWE remained according to the inclusion and exclusion criterion. These included 1,049, 309, and 669 individual records of patients receiving VPA, CBZ, and OXC therapies, respectively (Figure 1). The clinical characteristics of the study participants are presented in Table 1.

### Analysis of the Valproic Acid Group

**FIGURE 2:**
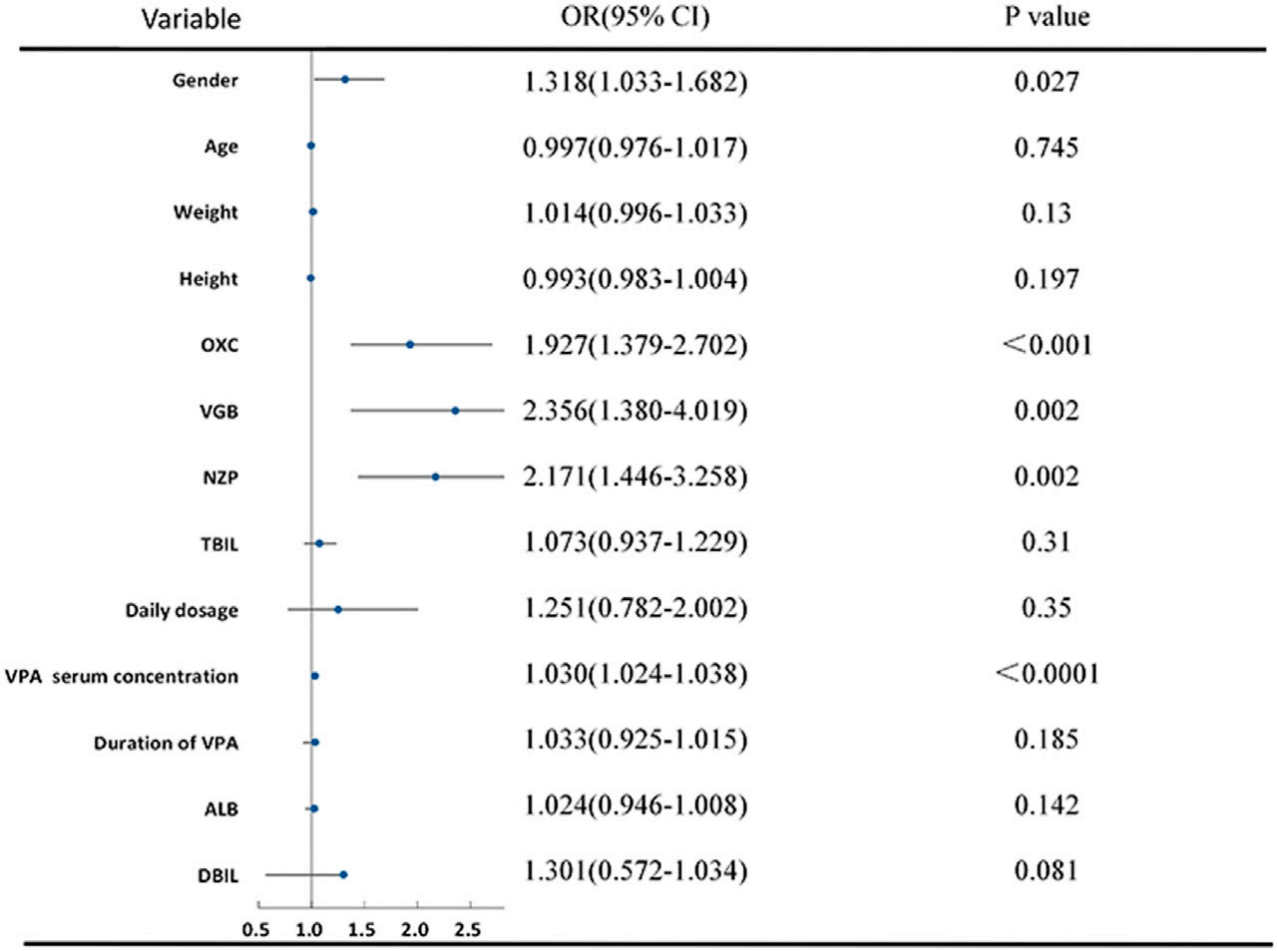
Ordinal logistic regression analysis of predictors of VPA efficacy.

The VPA group, dosage form, sex, seizure type, and its combination with OXC, VGB and NZP were included in the multivariable ordinal logistic regression analysis. Pearson χ2 test revealed significant differences between the groups (*p* < 0.05) (Table 2). In addition, according to the homogeneity test of variance (*p* > 0.1), total bilirubin (TBil) and direct bilirubin (DBIL) were included by ANOVA. However, age, height, weight, daily dosage, the serum concentration of VPA, and duration of VPA administration were included after the Kruskal-Wallis test (*P* < 0.05) (Figure 2). There was no significant difference in the test of parallel lines of ordinal logistic regression (*χ* ^*2*^ = 46.25, *p* = 0.99). Seizure type, dosage form, and visit times were treated as dummy variables in the regression analysis. VPA showed a higher efficacy in female compared to male (OR 1.318 [1.033–1.682], *p* = 0.027). With the increase of every unit (1 mg/L) of VPA serum concentration, there is 1.03 times possibility of improvement of efficacy (OR 1.030 [1.024–1.037], *p* < 0.0001). Compared to its use as a single agent, VPA in combination with OXC (OR 1.93 [1.38–2.70], *p* < 0.001), VGB (OR 2.36 [1.38–2.70], *p* = 0.002), or NZP (OR 2.17 [1.45–3.26], *p* = 0.002) exhibited a significantly better efficacy. Patients who attended the pharmacists’ educational classes three (OR 1.557 [1.067–2.270], *p* = 0.021) or four times (OR 1.71 [1.019–2.852], *p* = 0.042) showed a significant improvement in seizure reduction compared to those who were visiting the clinic for the first time. VPA also showed higher efficacy in patients with focal (OR 2.270 [1.124–4.581], *p* = 0.022) and generalized (OR 2.560 [1.20–5.176], *p* = 0.009) seizures compared to those with focal and generalized seizures. The results of continuous and binary variables were showed in Figure 2, and multilevel variables’ were presented in Table 2.

**TABLE 2:** Correlation between dummy variables and AED efficacy by ordinal logistic regression.

	VPA			CBZ			OXC		
Variable	OR	P-value	95% CI	OR	P-value	95% CI	OR	P-value	95% CI
**Seizure type**									
Generalized & focal	1			No			1		
Generalized	2.56	0.009	1.27.5.18	No	No	No	0.83	0.699	0.32.2.13
Focal	2.27	0.022	1.12.4.58	No	No	No	2.93	0.033	1.09.7.85
Unknown	5.00	<0.0001	2.41.10.36	No	No	No	0.23	0.628	0.50.3.15
**Dosage form**									
Ordinary tablet	1			No			No		
Oral solution	1.29	0.379	0.73.2.25	No	No	No	No	No	No
Sustained release tablet	1.44	0.17	0.86.2.41	No	No	No	No	No	No
**Visit times of outpatient service**				No	No	No	No	No	No
Baseline (1)	1			1			1		
2	1.11	0.476	0.83.1.50	1.81	0.103	0.89.3.70	1.57	0.016	1.09.2.26
3	1.56	0.021	1.07.2.27	2.72	0.043	1.06.4.19	2.28	0.004	1.31.3.97
4	1.71	0.042	1.02.2.85	2.78	0.046	1.07.4.67	1.35	0.424	0.65.2.82
5	1.31	0.445	0.65.2.65	1.35	0.581	0.78.2.85	1.36	0.624	0.39.4.66
6	1.03	0.951	0.40.2.67	1.23	0.872	0.43.2.87	1.77	0.756	0.34.4.83

No: No significant differences existed between the groups in previous cross tabs with Chi-square of Independence analyses. VPA: Valproic acid; CBZ: Carbamazepine; OXC: Oxcarbazepine; AED: anti-epileptic drug.

### Analysis of the Carbamazepine Group

**FIGURE 3:**
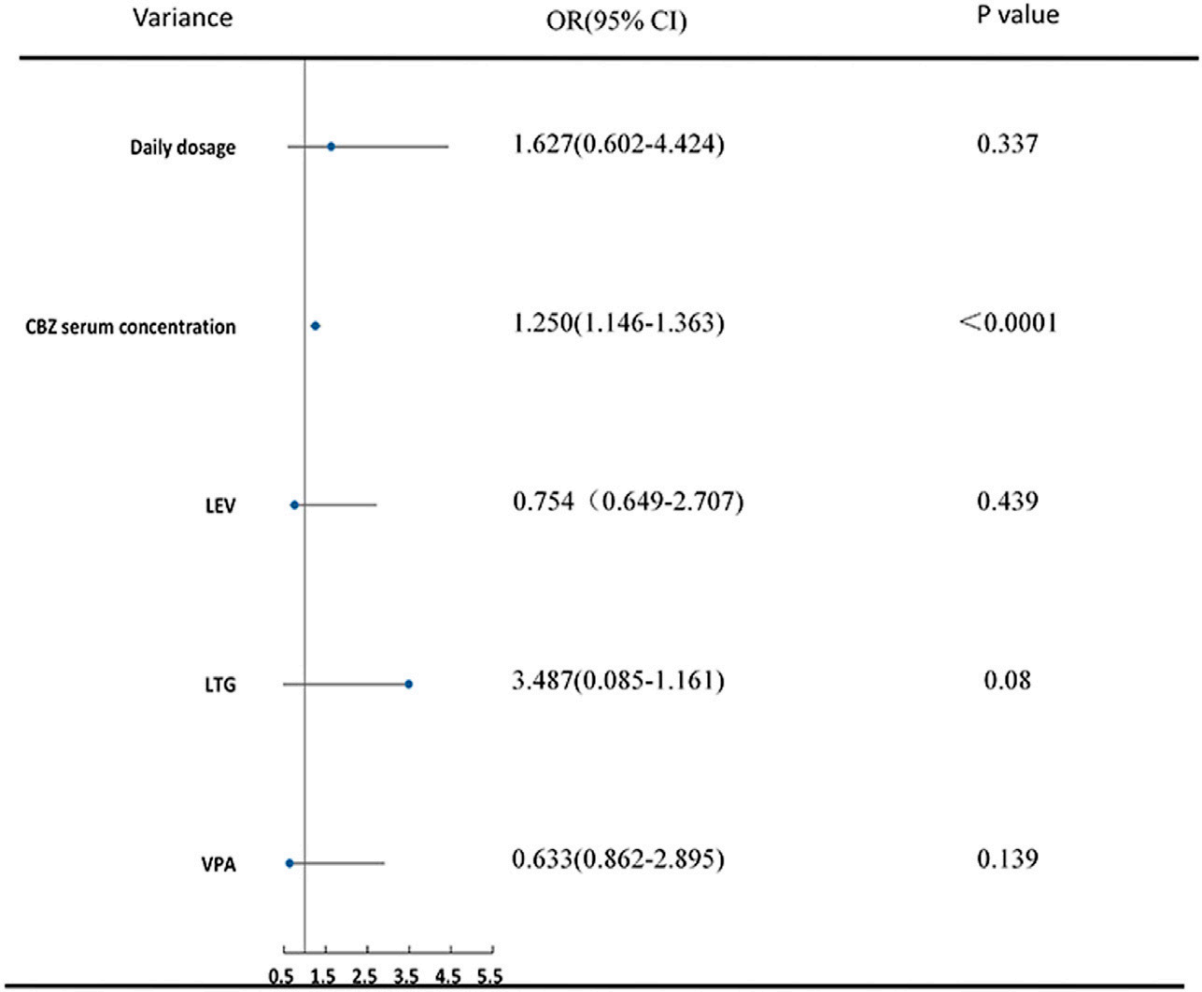
Ordinal logistic regression analysis of predictors of CBZ efficacy.

Using the same statistical methods as for the VPA group, serum concentrations of CBZ, daily dosage, visit times, and its combination with LEV, LTG and VPA were included in the final ordinal logistic regression. Higher CBZ serum concentrations (OR 1.250 [1.146–1.63], *p* < 0.0001) were indicative of better efficacy in PWE. Additionally, patients who received instructions from the pharmacists three (OR 2.721 [1.060–4.191], *p* = 0.043) or four (OR 2.883 [1.181–4.670], *p* = 0.046) times showed a significant improvement in seizure reduction compared to patients who were visiting the clinic for the first time (Table 2; Figure 3).

### Analysis of the Oxcarbazepine Group

**FIGURE 4:**
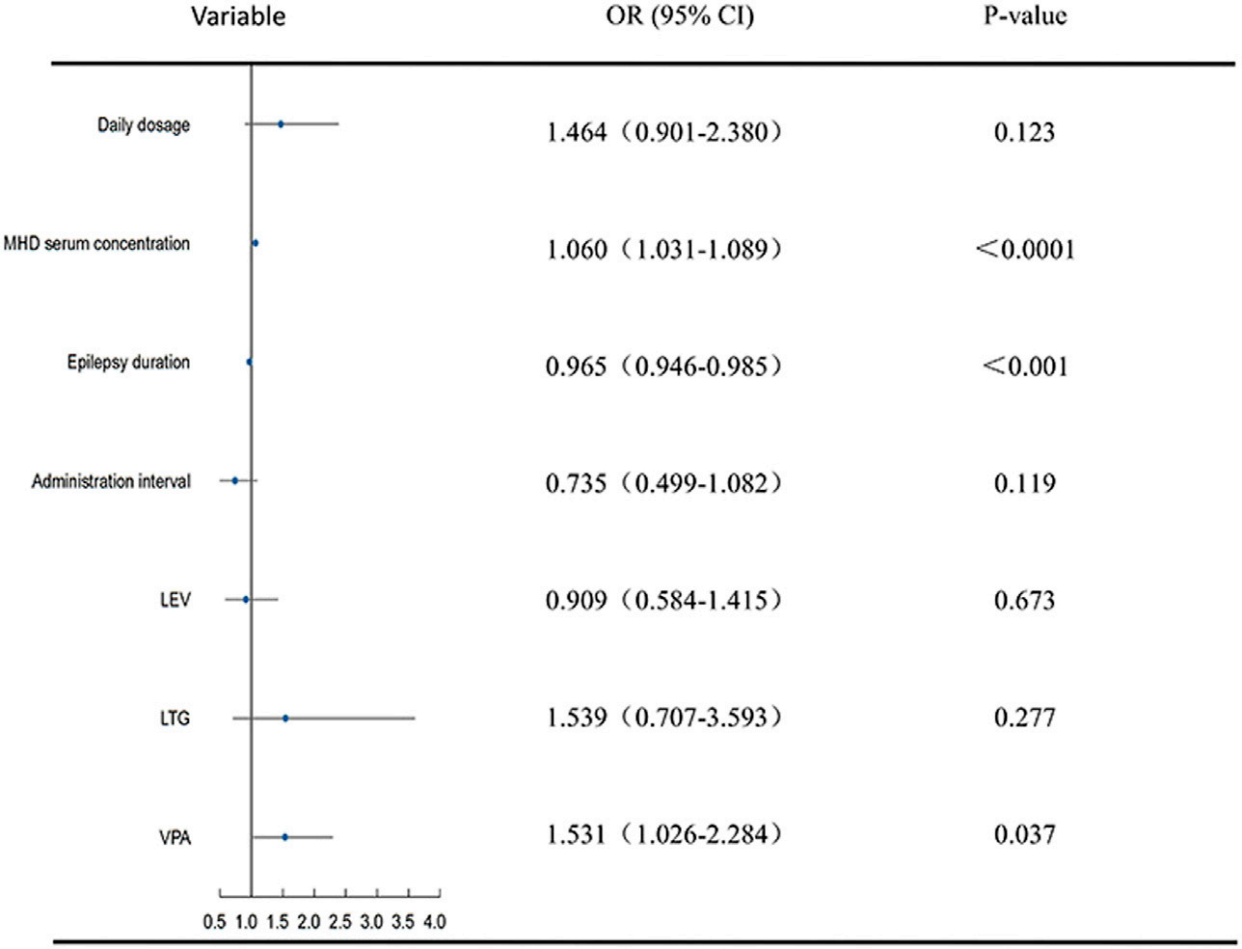
Ordinal logistic regression analysis of predictors of OXC efficacy.

Daily dose, dosage frequency, serum concentrations of MHD, duration of epilepsy, visit times, seizure type as well as the combination of OXC with LEV, LTG, and VPA were included in the ordinal logistic regression. The efficacy of OXC in improving seizure reduction was significantly correlated with its metabolite (MHD) serum concentration in statistical analysis (OR 1.060 [1.031–1.089], *p* < 0.0001), duration of epilepsy (OR 0.965 [0.946–0.984], *p* < 0.001), the second (OR 1.567 [1.087–2.259], *p* = 0.016) and third (OR 2.282 [1.310–3.971], *p* = 0.004) visits to the outpatient clinic, as well as its combination with VPA (OR 1.531 [1.026–2.824], *p* = 0.037) (Table 2; Figure 4).

### Efficacy Comparison of Valproic Acid, Carbamazepine and Oxcarbazepine With Combination of One Additional Drug

**FIGURE 5:**
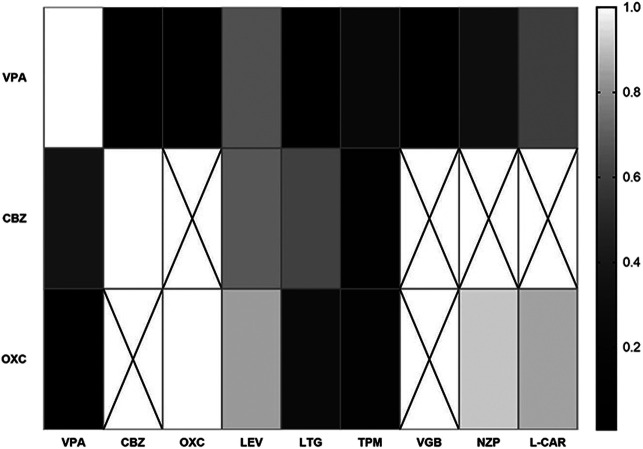
Correlations of certain combinations with single agent of VPA, CBZ and OXC.

Further validation of the ideal combination of VPA, CBZ and OXC was analyzed. The number of combinations which fewer than ten participants was deleted in this analysis. The efficacies of VPA/LEV (*p* = 0.660) and VPA/VGB (*p* = 0.006) were proved to be significantly better than a single VPA regimen. And OXC/VPA (*p* = 0.037) also significantly improved the efficacy compared to OXC monotherapy (Table 3). VPA/NZP showed no significant differences in the analysis. Therefore, it was excluded from the recommended combination after this validation step. The correlations of combinations of VPA, CBZ and OXC are showed in Figure 5 (all p values of samples were included except for no particular combination).

**TABLE 3:** Comparative efficacy of VPA, CBZ and OXC with additionally single drug combination.

AEDs treatment	No	^—^X_SR_	Z score	P Value
**VPA**	439	3.36		
VPA/LEV	178	3.21	−0.439	0.660
VPA/LTG	36	3.64	−1.928	0.054
VPA/TPM	58	3.46	−1.093	0.274
VPA/CBZ	26	2.85	−1.960	0.052
VPA/OXC	102	3.76	−0.2354	0.019*
VPA/VGB	25	3.94	−2.733	0.006*
VPA/L-CAR	49	3.45	-0.540	0.589
VPA/NZP	28	3.43	−0.956	0.339
**CBZ**	216	3.05		
CBZ/LEV	31	3.16	−0.415	0.678
CBZ/VPA	26	2.85	−0.848	0.397
**OXC**	447	3.18		
OXC/LEV	73	3.11	−0.210	0.833
OXC/VPA	102	3.76	−2.089	0.037*
OXC/LTG	19	2.74	1.122	0.262
OXC/TPM	15	2.67	−1.473	0.141

^—^X_SR:_ Mean of seizure reduction; *: statistical significance (p<0.05).

### Efficacy Comparison in Valproic Acid, Carbamazepine, and Oxcarbazepine for Monotherapy and Polytherapy

VPA, CBZ and OXC efficacy of monotherapy were compared with polytherapy. A single treatment of VPA (*p* = 0.037) or OXC (*p* = 0.013) is more effective than combining with other AEDs (the small data quantity of CBZ may result in non-significant differences between the two groups, *p* = 0.279).

### Adverse Effects Incidences Comparison in Valproic Acid, Carbamazepine, and Oxcarbazepine

AE categories were evaluated among outpatients who received monotherapy with VPA, CBZ, and OXC based on the PROs (The AEs were categorized based on their mechanism of action (Margolis et al., 2014)). AE incidences were not significantly reduced in OXC than VPA and CBZ except for psychiatric disorder (*p* = 0.002) and nervous system (<0.0001) disorders. These AEs were also found in the 3 AEDs (occurrence rate in VPA or CBZ was less than OXC) (Table 4).

**TABLE 4:** Adverse drug effect (AEs) among PWE on monotherapy of AEDs.

Classification of AEs	Number of ADRs among PWE on monotherapy		
Category of AEDs	VPA(*N* = 439) n (%)	CBZ (*N* = 216) n (%)	OXC (*N* = 447) n (%)	P value
Psychiatric disorders	23 (5.2)	9 (4.2)	5 (1.1)	0.002
Nervous system disorders	23 (5.2)	46 (21.3)	82 (18.3)	<0.0001
Skin and subcutaneous tissue disorders	7 (1.6)	5 (2.3)	7 (1.6)	0.758
Metabolism and nutrition disorders	13 (3.0)	2 (0.9)	10 (22.4)	0.258
Gastrointestinal disorders	38 (8.7)	14 (6.4)	23 (5.1)	0.114
Eye disorders	0 (0)	2 (0.9)	8 (1.8)	—
Reproductive system and breast disorders	1 (0.2)	2 (0.9)	3 (0.7)	—
Ear and labyrinth disorders	1 (0.2)	0 (0)	2 (0.4)	—
Hepatobiliary disorders	2 (0.4)	0 (0)	0 (0)	—
Blood and lymphatic system disorders	1 (0.2)	0 (0)	1 (0.2)	—

VPA: Valproic acid; CBZ: Carbamazepine; OXC: Oxcarbazepine; AED: anti-epileptic drug; PWE: patients with epilepsy.

## Discussion

Based on the clinical records of 3,157 outpatients with epilepsy, this study evaluates the factors that influence the efficacy of three AEDs. The AEDs were VPA, CBZ, and OXC. One of the significant factors that influenced the efficacy of VPA was the patient’s sex. VPA resulted in better seizure reduction in the female when compared to male. Several studies have found that the pharmacokinetic (PK) and pharmacodynamic (PD) properties of AEDs are influenced by sex (Schwartz, 2007). Lower VPA clearance of in females has been observed in several studies (Yukawa et al., 1997; Ogusu et al., 2014). VPA is mainly metabolized via three metabolize routes including glucuronidation, CYP450 enzymes, and β-oxidation (Perucca, 2006; Ghodke-Puranik et al., 2013). The lower UDP-glucuronosyltransferases activity in females leads to slower clearance of VPA (Franconi et al., 2007). Furthermore, a PK analysis has also revealed that sex has a significant effect on the disposition parameters by different hepatobiliary transfers of VPA, which resulted in a 2.1-fold higher reabsorbed fraction in women than in men after a single VPA dose. An inductive effect of ethinylestradiol on glucuronosyltransferase activity may be the main reason for that (Ibarra et al., 2013). However, few studies based on large real-world data have indicated the gender differences in VPA efficacy. Therefore, more reliable evidence on the sex-related clinical efficacy of AEDs is still required.

Consistent with other studies (Ghodke-Puranik et al., 2013), we also validated that the serum concentrations of VPA and CBZ showed a significant correlation with their respective efficacies. As well, OXC efficacies were also correlated to MHD serum concentrations (May et al., 2003). It seems that our research contained a larger sample size and much broader considerations of factors compared to other studies (Chen et al., 2015; Zhu et al., 2018). Since TDM of the total serum concentrations of AEDs remains one of the primary methods for measuring drug efficacy indirectly (Cook et al., 2016), it is not enough for researchers only focus on serum concentration. Jacob et al. claim that standardized studies should be designed to assess concentration–efficacy–toxicity relationships when AEDs are urgently required (Jacob and Nair, 2016). As the updated guidelines suggest, regular TDM in adults and children is not recommended and should be carried out only if clinically indicated (Excellence(NICE), 2012). Our longitudinal observational study provided the significantly correlated factors with AEDs efficacy based on real-world clinical data in PWE. However, further bigger and better randomized, controlled studies are required to confirm our findings.

Furthermore, based on the ordinal logistic regression analysis, we found that VPA is more effective in controlling generalized, focal, or unclassifiable seizures than compared to focal and generalized seizures. On the other hand, OXC shows better clinical performance in patients with focal seizures compared to those with focal and generalized seizures. It is well-acknowledged that VPA is a good choice, particularly for generalized epilepsy syndromes (Beghi and Giussani, 2019). OXC, as a sodium channel modulator, is appropriate for focal epilepsies (Thijs et al., 2019). Since ILAE has combined generalized and focal epilepsies into a new category for patients presenting with both seizure types (Scheffer et al., 2016), this is the first study of AEDs to our knowledge based on this new classification.

**FIGURE 6:**
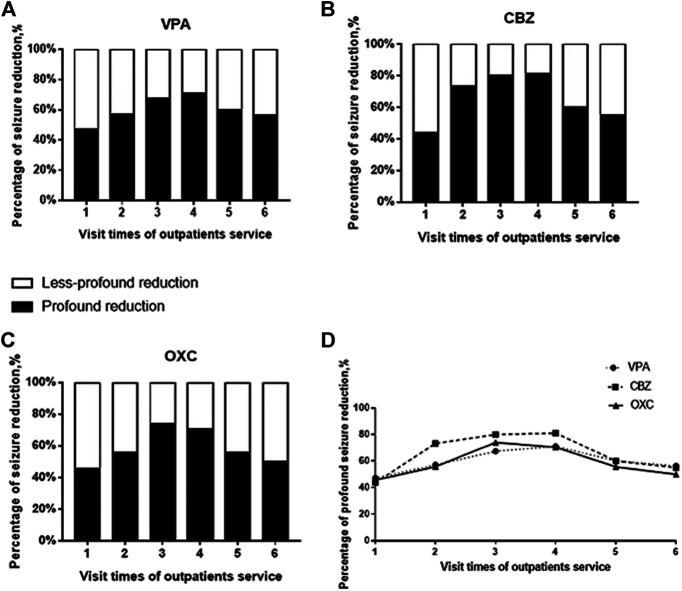
Probability of AEDs having profound efficacy based on the number of outpatient visits.

We also found that seizures decreased significantly after the patients’ first to the third visit to the outpatient services, suggesting that the education provided by the pharmacists and the individual adjustment of AED dosage by the clinicians help in improve the treatment outcomes (Ma et al., 2019). Due to an overall improvement in the control of epilepsy, at least in China, the tendency of PWE to return to the clinics is declining. As a result, the proportion of drug-resistant PWE is likely higher during the fourth and subsequent visits. This could account for the lack of significant difference in the efficacy of AEDs in patients making the initial and fourth or fifth visits even though we provided the same services. We have used histograms and line diagrams to present these differences based on profound (≥75%) and less-profound (<75%) reduction in AED efficacy compared to the baseline (Nabbout et al., 2019) (Figure 6). Unlike some other pre-to post-intervention studies (Chen et al., 2013; Tang et al., 2014), we recorded the efficacy process after every pharmacist education instead of the result. As the tendency in Figure 6 shows, education by pharmacists only benefit for the seizure reduction in the first three times of visits. Since excessive medical education may not provide help for PWE, it inspires us that suitable and efficient pharmacists’ educational service need to be established.

Additionally, our analysis indicated that seizure frequency decreased significantly when VPA was combined with OXC or VGB compared to when it was used alone. Likewise, OXC showed significantly better efficacy in combination with VPA, compared to when used as a monotherapy. Given that there are limited data to support other synergistic AED combinations, our study may provide a reference to some extent. Increasing evidence showes that unrestrained polytherapy could even actually aggravate epilepsy in some patients (Reynolds et al., 2012). A previous study has claimed that there is much concern in the notion that certain AED combinations may be advantageous. Still there is only limited human data in support of specific combinations. Currently. solid human data exists only for the VPA and LTG combination (Abou-Khalil, 2017), supported by several studies (Poolos et al., 2012). Nicholas et al. analyzed some AED combinations in 148 patients. However, their study did not include OXC. We have found that the ideal combination is OXC-VPA when compared to each monotherapy, This may be a direction for the follow-up researches. Although our analysis found no significant difference in the efficacy of VPA + LTG combination and VPA alone. It is worth noticing that the combination of VPA/LTG also showed marginal significance (0.05 ≤ *P*<0.1) in Table 2 (*p* = 0.054), an insufficient sample size of LTG-VPA may be the reason. Therefore, VPA/LTG is likely an ideal combination, which may need further research. On the contrary, VPA combine with CBZ showed the marginal significance of worse efficacy than VPA monotherapy (Table 2), which may be because of the drug interaction. Jiao, et al. studied that concomitant administration of valproic acid and carbamazepine resulted in a 36% increase in valproic acid clearance (Jiao et al., 2004), which could be the reason. Besides, Charles L. P et al. found that no statistical differences between the CBZ-VPA combination and single-agent of CBZ in reducing of seizure frequencies (Deckers et al., 2001), which is consistent with our result. Moreover, Edward et al. raised a question that at in those minority of patients in whom seizure control improves with an additional AED, it is usually unclear whether improvement is due to the combination or to the second AED (Reynolds et al., 2012). Our study supported the former viewpoint that the improvement of efficacy derives from the synergistic effect of certain polytherapy. While there are several studies on the PK interactions in AEDs (Perucca, 2006; Zaccara and Perucca, 2014) the PD interactions between AEDs and other drugs are poorly characterized (Perucca, 2006). There are few studies carried out for the synergistic effect of non-antiepileptic drugs when combined with AEDs. Our study screened the first and second widely used non-AED drugs and found these 3 AEDs neither combined with NZP nor with L-CAR effectively reducing seizure frequency.

We also categorized the records based on the use of VPA, CBZ, and OXC for monotherapy and polytherapy (polytherapy were regarded as a whole, which means a combination with at least one AED without separate analysis in pairwise of each AEDs-combinations here) (Table 5). A single VPA or OXC treatment is more effective than general polytherapy (the small data quantity of CBZ may result in non-significant differences between the two groups). The result may explain the previous view that there is little evidence of polytherapy’s advantages over monotherapy (Reynolds et al., 1981). These findings are also consistent with those of a 30 years longitudinal cohort study, which reported that 46% (820) of the patients remained seizure-free for 1 year or longer with the first AED, while only an additional 18% patients became seizure-free after a change in treatment, despite increasing use of second- and third-generation AEDs (Chen et al., 2018). Therefore, despite we have figured out some kinds of polytherapy of AEDs in this 4 years-observational study which can improve the efficacy, such as OXC-VPA, we acknowledge that the superiority of monotherapy over polytherapy in the long duration of medication. Several studies have also reviewed the potential benefits of reducing polypharmacy and have, surprisingly, seen improvement in seizure control when polytherapy was changed to monotherapy (Abou-Khalil, 2017). Monotherapy remains the standard initial line of therapy for epilepsy. However, an ideal synergistic combination of AEDs has supra-additive efficacy and infra-additive side effects (Kralj-Hans et al., 2014), which should considered when selecting AED combinations for clinical treatment of epilepsy. As the best way to combine AEDs for PWE requiring polytherapy remain a subject of much debate (Stephen et al., 2012), clinical researches for exploring the ideal AED combinations is necessary, especially for newer AED agents. Our study, which was based on clinical real-world and providing the validated evidences of ideal AEDs-combinations, may help choose the effective regimen of AEDs.

**TABLE 5:** AED efficacy in monotherapy and polytherapy groups.

Category of AED	Mono-therapy (n)	Poly-therapy (n)	Mono-therapy (mean rank)	Poly-therapy (mean rank)	Z value	P value
VPA	439	619	546.80	509.30	−2.803	0.037
CBZ	216	93	158.49	149.90	−1.083	0.279
OXC	447	222	347.69	309.46	−2.496	0.013

VPA: Valproic acid; CBZ: Carbamazepine; OXC: Oxcarbazepine; AED: anti-epileptic drug.

Outpatients were chosen for the target population because, when compared to the inpatients, they received simpler drug-combinations and fewer non-drug treatments, especially surgery. Although age is considered to be related to compliance with AED use (Modi et al., 2011), we found no differences between the various age groups during the preprocessing of data for analysis. We, therefore, did not regard age as hierarchical data.

The goal of epilepsy treatment is to control seizures without any AEs. However, approximately 88% of the patients often experience AEs from the AEDs (Lamberink et al., 2017). We also evaluated AE categories among outpatients who received monotherapy with VPA, CBZ, and OXC. OXC, a second-generation AED, showed almost no significant difference compared to the conventional AEDs (VPA, CBZ). Although the second generation of AEDs is considered superior to conventional AEDs, with fewer AEs and better efficacy, there is no clear evidence to support this assumption. Our findings are consistent with those of a recent cross-sectional study that demonstrated that newer AEDs are associated with similar adverse drug reactions to conventional AEDs (Kumar et al., 2020). Therefore, when selecting an AED, clinicians should focus on its efficacy in addition to the safety data. Several studies have shown that the efficacy of the second generation of AEDs, both as monotherapy or combination therapy, is similar to that of conventional AEDs. Whether the newer drugs improve the overall prognosis of epilepsy, remains controversial (Loscher and Schmidt, 2011; Beghi and Giussani, 2019).

Since the data derived from clinical real-world observation, this study may be affect by certain factors. Several limitations warrant mention. First, a lot of records were excluded due to missing outcomes. Noncooperation of patients and unevaluable seizure frequency was the main reason for that in real-world data collection. However, the data was not excluded on purpose, but it would affect the result to some extent. Second, potential confounders may exist in characteristics of different patients, which cannot be controlled completely for inherences of the clinical observational study. Although we tried our best to ensure the accuracy of baseline frequency by both of initial electronically medical stored data and paper records along with careful inquires of patients/guardians, the bias may could not be eliminated. One reason is the unobservable or imperceptible seizure of patients. The other reason is observed omissions of seizure frequency. Although the total number of samples were adequate, because of the preferences of monotherapy as well as guidelines for combination medication by clinicians, some certain combinations did not have a large enough sample. This may have caused statistical errors. Finally, we did not consider factors such as genetic polymorphism and diet style of patients, that may also affect AED efficacy and adverse effect. Further researches into these factors is needed.

## Conclusion

The serum concentrations of VPA and CBZ may positively affect their efficacies. OXC efficacies also correlate to MHD serum concentrations. VPA is more effective in reducing seizures in females compared to males. Additionally, VPA is more effective for focal, generalized, and unclassifiable seizures than for focal and generalized ones. The efficacy of OXC correlates with the duration of epilepsy. Combinations of VPA with OXC/VGB also have higher efficacies compared to VPA alone. OXC-VPA, likewise, is an ideal combination too. Pharmacists’ education combined with the dynamic, individualized treatment provided by the clinicians is likely to improve seizure control in PWE. Monotherapy of VPA and OXC shows a better efficacy than polytherapy as whole. And there were no significant differences between most categories of adverse effects for the investigated AEDs.

## Data Availability

The raw/processed data required to reproduce these findings cannot beshared at this time as the data also forms part of an ongoing study.
